# Lessons Learned: Social and Ethical Issues Related to Clinical Implementation of Blood‐Based Biomarkers for Alzheimer’s Disease

**DOI:** 10.1002/alz.089279

**Published:** 2025-01-09

**Authors:** Fred B. Ketchum

**Affiliations:** ^1^ Neurology, University of Wisconsin‐Madison School of Medicine and Public Health, Madison, WI USA

## Abstract

**Background:**

Integrating blood biomarker testing for Alzheimer’s Disease (AD) into clinical practice has the potential to transform AD care by enabling broadly accessible and accurate diagnosis, more precise prognostication, and timely initiation of disease‐modifying therapy. While there are several scientific challenges to implementing blood biomarkers (e.g. validity in different populations or the impact of comorbidities), the range of ethical and social issues that must be addressed to ensure that blood biomarker testing is appropriately implemented has received less attention.

**Methods:**

Incorporating current data through a critical literature review focused on ethical and social aspects of AD biomarker and specifically blood biomarker testing, disclosure, and implementation, we extracted arguments, grouped them according to bioethical principles, and developed a framework to categorize ethical and social issues around blood biomarkers across clinical settings.

**Results:**

Issues identified were related to the four basic principles of bioethics: respect for autonomy, beneficence, non‐maleficence, and justice; and were informed by social norms including standards of professional practice and definitions of health and disease. The main ethical and social issues were related to: clinical and personal utility of testing; prognostic uncertainty of results; the expansion of disease categories; preferences among patients, caregivers, and families regarding testing and disclosure; facilitating positive psychological outcomes; enabling positive health‐related behavior changes; potential for stigma, privacy, and discrimination; decision‐making and communication in diverse clinical settings and patient populations; effective use of diagnostic and treatment resources; and ensuring equitable access to testing and care.

**Conclusion:**

Implementing blood biomarkers in clinical practice in an ethically and socially responsible manner will require 1) maximizing benefits while minimizing risks; 2) informing and empowering individuals to make decisions about their brain health; and 3) ensuring testing is available equitably. Ethical and social issues may be different before versus after testing, and while some issues are similar to those encountered in AD diagnosis more generally, others are specific to blood biomarkers. Key areas in which data are needed include approaches to minimizing negative psychosocial outcomes, determining clear distinctions between disease and at risk‐states, and promoting effective communication, education, and equitable access.

